# Two-Stage Plasma-Thermal Nitridation Processes for the Production of Aluminum Nitride Powders from Aluminum Powders

**DOI:** 10.3390/ma12030359

**Published:** 2019-01-24

**Authors:** Mei-Chen Sung, Ya-Fen Wang, Shang-Che Chen, Cheng-Hsien Tsai

**Affiliations:** 1 Department of Chemical and Materials Engineering, National Kaohsiung University of Science and Technology, Kaohsiung 807, Taiwan; k12m34333@gmail.com (M.C.S.); w7866988@hotmail.com (S.C.C.); 2 Department of Environmental Engineering, Chung Yuan Christian University, Chung-Li 320, Taiwan; yfwang@cycu.edu.tw

**Keywords:** plasma nitridation, thermal nitridation, aluminum nitride, pre-synthesis, agglomeration

## Abstract

The synthesis of aluminum nitride (AlN) powders is traditionally done via the thermal nitridation process, in which the reaction temperature reaches as high as 960 °C, with more than several hours of reaction time. Moreover, the occurrence of agglomeration in melting Al particles results in poor AlN quality and a low efficiency of nitridation. In this study, an atmosphere-pressure microwave-plasma preceded the pre-synthesis process. This process operates at 550 °C for 2–10 min with the addition of NH_4_Cl (Al: NH_4_Cl = 1:1) for generating a hard AlN shell to avoid the flow and aggregation of the melting Al metals. Then, the mass production of AlN powders by the thermal nitridation process can be carried out by rapidly elevating the reaction temperature (heating rate of 15 °C/min) until 1050 °C is reached. X-Ray Diffractometer (XRD) crystal analysis shows that without the peak, Al metals can be observed by synthesizing AlN via plasma nitridation (at 550 °C for 2 min, Al: NH_4_Cl = 1:1), followed by thermal nitridation (at 950 °C for 1 h). Moreover, SEM images show that well-dispersed AlN powders without agglomeration were produced. Additionally, the particle size of the produced AlN powder (usually < 1 μm) tends to be reduced from 2–5 μm (Al powders), resulting in a more efficient synthesizing process (lower reaction temperature, shorter reaction time) for mass production.

## 1. Introduction

Aluminum nitride (AlN) is a superior ceramic material for packaging and microelectrical substrates due to its high wear resistance, high thermal conductivity (200–320 Wm^−1^K^−1^), low thermal expansion coefficient (2.6–5.5 × 10^−6^ K^−1^), high volume resistivity (10^11^ Ωm), low permittivity, non-toxicity, and wide band gap (6.2 eV) [1,2]. It has been successfully applied to high density packaging, large-scale integrated circuits, and wide-bandgap power amplifiers in the semiconductor industry, military, and other industrial applications.

Many techniques have been developed in the past decades for synthesizing AlN powders. Several approaches have been developed such as the solid-solid reaction method via a carbothermal reduction method that reduces Al_2_O_3_ or Al_2_SiO_5_ with carbon in an N_2_ environment at 1650–1800 °C [3,4], the liquid–liquid reaction via organometallic precursors (such as [(CH_3_)_2_AlNH_2_]_3_) reacting with liquid NH_3_ at 1000 °C [3], the chemical vapor deposition reaction of gaseous AlCl_3_ and gaseous N_2_/NH_3_ at 1200–1400 °C [3,5], and the liquid–gas reaction of metal Al powder via direct thermal nitridation with N_2_ gas at 1000–1400 °C [3,6]. Self-propagating high-temperature synthesis (or combustion synthesis) via ignition of Al and NH_4_*X* (*X* = F, Cl, Br) compacted in N_2_ at 1000–1200 °C and at high pressure was also proposed [7,8]. The application of plasma techniques for the synthesis of AlN powders has been introduced by various researchers; such as direct-current (DC) arc plasma [1,9,10,11], transferred arc plasma [12,13], radio-frequency plasma [14,15,16], DC pulsed wire discharge [17,18], and microwave plasma [19]. All of these synthesis techniques require high reaction temperatures (1000–1100 °C) and long reaction times (1–5 h). However, due to high-temperature operation, the aggregation of Al powders is always a concern, although adding NH_4_Cl as an additive can effectively reduce the occurrence of agglomeration [7,20]. In addition, mechanical alloying under NH_3_ flow by milling aluminum powder at room temperature and a subsequent thermal treatment below the melting temperature of Al could be carried out and lead to a significant amount of nanocrystalline AlN [21].

Among all these applicable AlN synthesis techniques, the most commonly used technique is the thermal nitridation method due to its simplicity for mass production, in which the Al powders are heated up above the Al melting point (660 °C) for an extended period of time. As a result, the grain and agglomerate particle size of AlN grow with the increasing reaction temperature due to the coarseness of the starting Al powder [22]. The agglomerate particles gradually increase with temperature, from 3.78 μm at 1000 °C to 3.90 μm at 1100 °C, before finally reaching 5.40 μm at 1200 °C. Moreover, the coagulation of melted Al particles during the nitridation process will inhibit the nitrogen gas diffusion into the core of unreacted Al particles because nitrogen has very low solubility in liquid Al [6]. Though adding NH_4_Cl powders could reduce agglomeration, the process usually requires a long reaction time (3–6 h) and a high reaction temperature (1000–1500 °C). In addition, a post-calcination treatment due to high N_2_ diffusion resistance is usually mandatory. As a consequence, it results in a lower nitridation rate, poorer AlN quality, and higher cost. 

The other approach is the application of plasma technology, by which sufficient energy can be quickly generated to excite nitrogen molecules or atoms so that the active nitrogen-containing compounds can rapidly react with the Al atoms to form AlN. Consequently, it can effectively shorten the reaction time and increase the conversion rate [1,9,10,11]. However, implementation of this plasma technique is extremely costly, which also limits its application for mass production. 

This study proposes a new process by adopting atmosphere-pressure microwave plasma as a pre-synthesis process, followed by the traditional thermal nitridation process. The purpose of the plasma pre-synthesis process is to quickly excite nitrogen atoms, nitrogen molecules, and nitrogen ions so that they can easily react with the aluminum atoms to form a thin aluminum nitride shell on the surface, which would prevent the flow of melting aluminum liquid and eliminate the aggregation of Al powders. The short-time plasma pre-synthesis process is then followed by the traditional thermal nitridation process, which can reduce the cost and enhance the applicability for mass production. 

Since this newly proposed process takes advantages of both plasma and traditional thermal nitridation techniques, it is expected that the newly combined process can be applied for mass production with better quality AlN achieved at a lower cost. 

## 2. Materials and Methods 

In this study, a two-stage synthesis process for fabricating AlN powders was carried out. In the first stage, an Al (ThinTech Materials Tech. Co., Ltd., Kaohsiung, Taiwan purity: 99.8%; particle size: 2–5 μm)/NH_4_Cl (Sigma-Aldrich, Saint Louis, MO purity: 99.99%; particles sizes: 1–4 μm) ingot (thickness ~5 mm) was prepared at 2000 psi by a tablet machine and placed in the microwave discharge effluents (N_2_ plasma) at a low temperature (550 °C) to form an AlN shell layer for 2–10 min with an applied power of 1200 W. Prior to the synthesis, the chamber was flushed with high-purity N_2_ to remove residual oxygen and steam. The flow rate of gas was regulated by a mass flow controller. The axial gas and swirl gas were both N_2_, with flow rates of 3 and 9 standard liters per minute (slm), respectively. 

**Figure 1:**
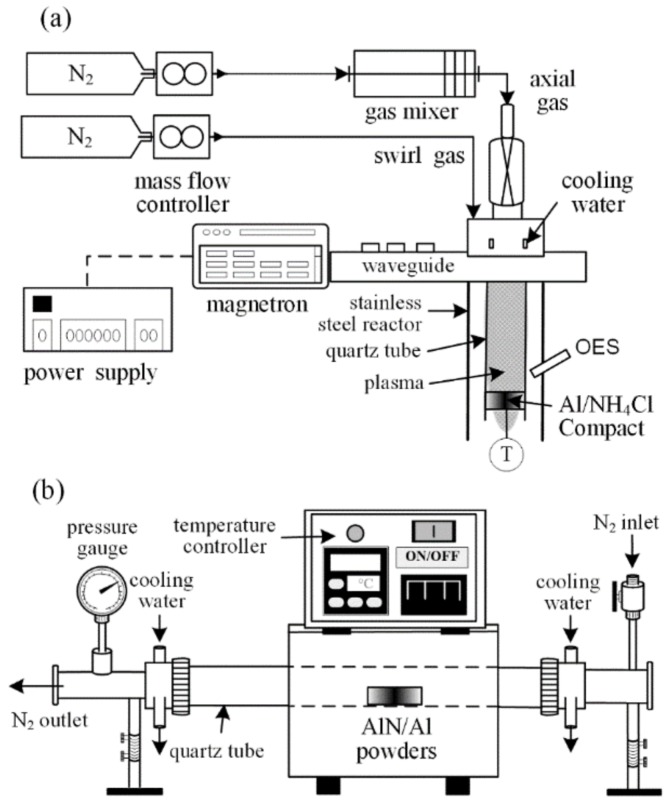
Schematic of synthesis apparatus (**a**) plasma-nitridation system, (**b**) thermal-nitridation system.

In the first stage, the microwave plasma-nitridation system (Figure 1a) used in this study was similar to that used in a previous study [19]. The main apparatus was assembled in a commercially available magnetron (YJ-1600, National Electronics, Hampshire, UK, 2.45 GHz) with a maximum stationary power of 5 kW. A quartz tube intersected the waveguide (WR340, ASTEX, Reichelsheim, Germany), and a resonator was placed perpendicular to it. Microwaves were emitted in continuous-wave mode and passed through a circulator and a waveguide with three stub-tuners before reaching the cavity. At the cavity, an arc ignited the plasma when nitrogen was introduced at atmospheric-pressure.

In the second stage, a relatively high temperature (850–1050 °C, heating rate of 15 °C/min) in a high temperature furnace (Figure 1b) was held for synthesizing AlN powders for 1 h with a total N_2_ flow rate of 1 slm.

The crystal structure of the synthesized AlN powders was determined using X-ray diffraction (XRD, RINT-2000, Rigaku, Texas, USA) with CuKα radiation in a scan range of 15°–85° (2θ). The whole ingot of prepared AlN/Al samples was set in the holder of XRD to analysis. The thermal characteristics of raw Al powders and the prepared powders were determined using thermogravimetric analysis (TGA, SDT Q600, TA Instruments, DE, USA) by heating the powders from room temperature to 1400 °C (held 1 h) at a heating rate of 10 °C/min in nitrogen gas with a flow rate of 100 ml/min. To prepare the TGA sample, the ingot was grinded first, and 20 mg of mixed powders sample were analyzed. The powders were examined using scanning electron microscopy (SEM, S3000N, Hitachi, Toronto, Canada) to determine their particle sizes and morphologies. Optical emission spectroscopy (OES, HR 4000CG, Ocean Optics, Inc., FL, USA) was used to measure the active species involved in the synthesis of AlN in the discharge zone. 

## 3. Results and Discussion

### 3.1. Effects of NH_4_Cl and Temperature on the Size and Dispersibility of Products by the Thermal Nitridation Process

**Figure 2:**
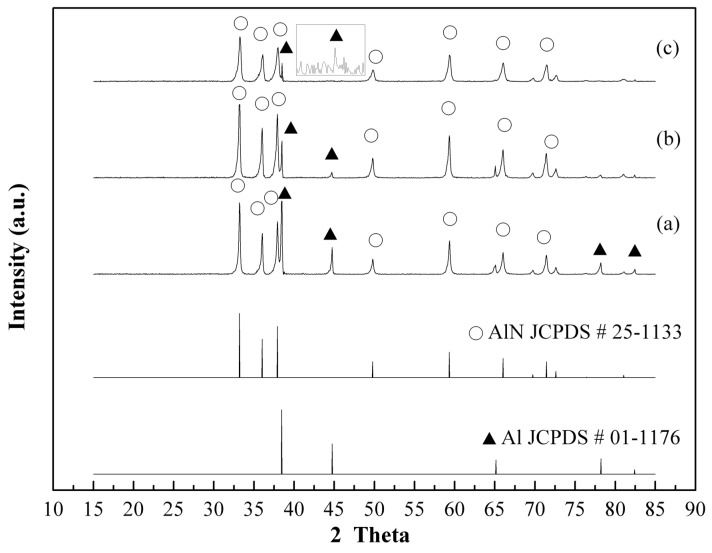
XRD patterns of AlN/Al powders produced by adding various NH_4_Cl contents in Al powders via thermal nitridation process at 850 °C (heating rate: 15 °C/min, held for 1 h) (**a**) 0%, (**b**) 30%, (**c**) 50%.

Based on the XRD diffraction patterns of aluminum nitride (aluminum nitride, JCPDS No. 25-1133) and Al (aluminum, JCPDS No. 01-1176), Figure 2a shows the XRD patterns of AlN powders using a thermal nitridation technique synthesized without adding NH_4_Cl (0%) at a heating rate of 15 °C/min from room temperature to 850 °C and held for 1 h. The characteristic peak intensity ratio, AlN (2θ = 33.2)/Al (2θ = 44.8), was only 2.7 due to a large amount of Al powders not being converted into AlN, which stemmed from the agglomeration of molten Al powders when the temperature was above the melting point (660 °C). The previous study also demonstrated the aggregation of molten mixtures of Al and AlN powders at a higher nitridation temperature [22].

With the addition of NH_4_Cl powders into the Al powders, solid NH_4_Cl sublimate and thermally decompose to HCl and NH_3_ at about 338 °C (NH_4_Cl_(s)_ = NH_3(g)_ + HCl_(g)_), resulting in a part of the as-prepared particles with irregular shapes, numerous porous structures, and a pore size similar to the size of NH_4_Cl powders [19]. Moreover, HCl can further react with Al to produce intermediate gaseous AlCl_3_ and H_2_ [6,23]. These pores can further provide a vapor reaction between AlCl_3_ and N_2_ to form AlN crystallization via the following reactions [2,22,24]:

Al_(s,l)_ + 3HCl_(g)_ = AlCl_3(g)_ + 3/2H_2(g)_

AlCl_3(g)_ + 1/2N_2(g)_ + 3/2H_2(g)_ = AlN_(s)_ + 3HCl_(g)_

This characteristic of NH_4_Cl makes it a great candidate to add into the nitridation process. It will not only prevent the aggregation or coalescence of molten Al but also promote the nitridation rate by facilitating the N-containing species to diffuse into the Al particles. To evaluate the effects of adding different amounts of NH_4_Cl powders in the nitridation process, various NH_4_Cl contents (30%, 50%) were added into Al powders for preparing AlN powders by the thermal nitridation process under the operating temperature of 850 °C for 1 hour. Figure 2b,c shows that the peak intensity ratio, AlN (2θ = 33.2)/Al (2θ = 44.8), increased to 15.6 and 38.4 by adding 30% and 50% NH_4_Cl, respectively. This implies that the conversion of Al increased with the increase of NH_4_Cl. However, the prepared AlN powders were still aggregated due to the synthesized temperature being higher than the melting point of the Al metals. Similar results were also found for the AlN powders synthesized by thermal nitridation techniques even at elevated reaction temperatures (950 °C, 1050 °C). 

**Figure 3:**
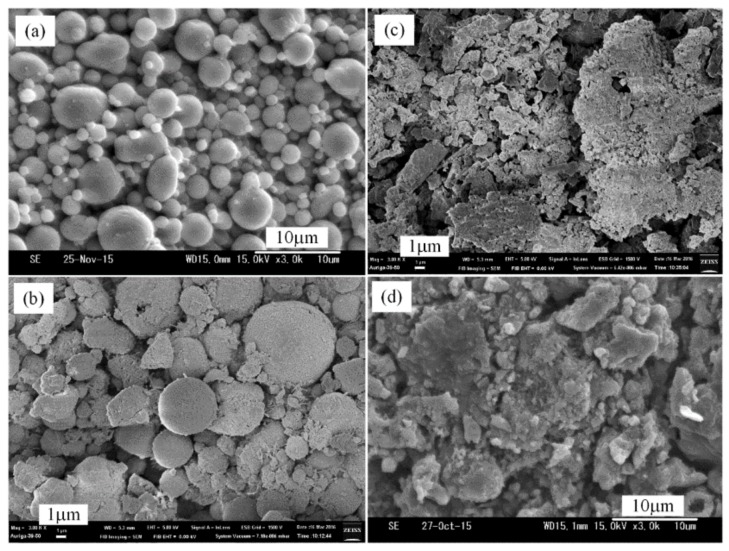
SEM micrographs (3000×) of original Al powders (**a**) and AlN/Al powders produced by thermal nitridation (Al/NH_4_Cl = 1:1, heating rate: 15 °C/min) at 850 °C (**b**), 950 °C (**c**), and 1050 °C (**d**).

Figure 3a shows the SEM micrograph of the original Al powder which is about 2–5 µm, as well as the synthesized AlN/Al powders produced by a thermal nitridation process under the following conditions: Al/NH_4_Cl ratio = 1:1 (50% NH_4_Cl), heating rate = 15 °C/min, and temperature elevated from room temperature to 850−1050 °C (Figure 3b–d) and held for 1.5 h. The SEM results showed that the synthesized AlN/Al particle size from thermal nitridation technique was found to be larger than that of the original Al powder. This is due to the occurrence of the agglomeration of melting Al powders at high temperatures, which is always a concern, although adding NH_4_Cl as an additive could reduce the occurrence of agglomeration, except for the big particles formed by the agglomeration of melting Al powders at high temperature. Some small and irregular particles are also found (Figure 3c) due to the nitridation of small Al particles (in Figure 3a) or the reaction between the species formed by the sublimation and decomposition of added NH_4_Cl particles and different size of Al particles. 

At 850 °C, the morphology of AlN/Al powder produced by the thermal nitridation technique is similar to that of the original aluminum powder, but larger sized (Figure 3b). It indicates that the original Al ball-shape powders begin to agglomerate with the temperature rising. When the operating temperature increases to 950 °C, the agglomeration phenomenon is even more significant as in the traditional thermal process shown in Figure 3c. As the temperature reaches 1050 °C (Figure 3d), the whole structure becomes irregular, with some particle sizes being greater than 10 µm with small particles attached on the surfaces. Obviously, the agglomeration of the AlN/Al powders is more serious with the increase of operating temperature when synthesizing AlN by traditional thermal nitridation processes. 

**Figure 4:**
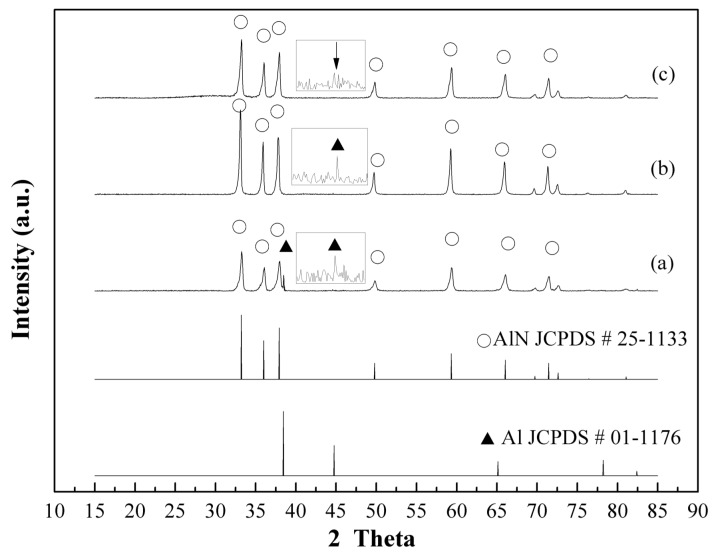
XRD patterns of AlN/Al powders prepared by thermal nitridation at (**a**) 850 °C (**b**) 950 °C (**c**) 1050 °C (heating rate: 15 °C/min, held for 1 h, Al/NH_4_Cl = 1/1).

Figure 4 shows the XRD patterns of as-prepared AlN/Al powders using traditional thermal nitridation techniques synthesized at elevated temperatures from 850–1050 °C and then held for 1 h. Obvious peaks of Al metal still existed from when the temperature was at 850 °C and 950 °C. The characteristic peak intensity ratio, AlN (2θ = 33.2°)/Al (2θ = 44.8°), increased from 38.4 to 59.1 as the temperature changed from 850 °C to 950 °C. However, at the reaction temperature of 1050 °C, Al peak intensity was very weak as a result of the significant agglomeration (Figure 4c) conducing only an AlN layer formed on the surface. 

To evaluate the AlN conversion, TGA data was used to evaluate the percentage of AlN after the nitridation processes. The AlN contents in the AlN/Al composite was calculated based on the following equation:

*X* = (151.85 – *Y*)/(151.85 – 100) × 100% (1)

where *X* is the content of AlN in the composite (%), *Y* is the final weight percentage obtained from TGA analysis, and 151.85 is the theoretical final weight percentage obtained from a TGA pattern for Al completely converting to AlN. 

Equation (1) was calculated by using TGA as well as the final weight percentage values reached—122.45%, 115.82%, and 114.90%—for the end nitridation temperatures being 850 °C, 950 °C, and 1050 °C, respectively, to indicate that the AlN content of the as-prepared products was only 56.7%, 69.5%, and 71.2%, respectively. The results showed that the AlN conversion is a little low even at 1050 °C nitridation temperature due to the coagulation of melted Al particles inhibits the diffusion of nitrogen passing through from AlN layer into the unreacted Al layer of the ingot, leading to the different patterns of AlN content between XRD and TGA analyses. 

### 3.2. Plasma Nitridation in the First Stage for Pre-Synthesizing AlN/Al Powders 

**Figure 5:**
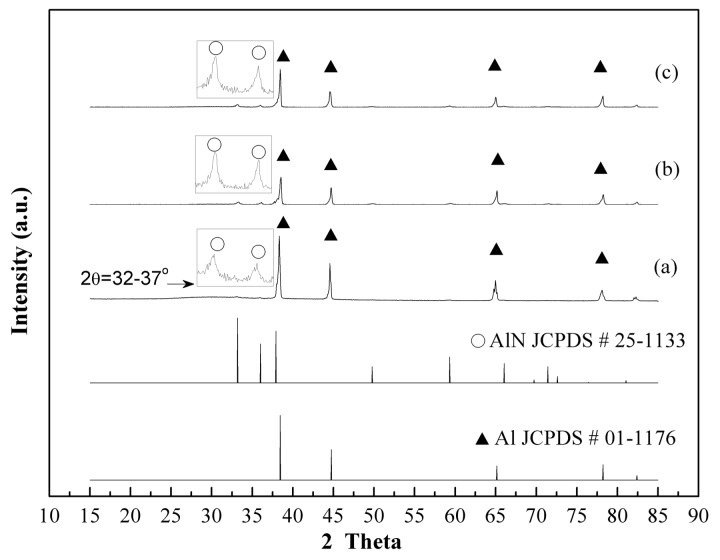
XRD patterns of prepared powders for plasma nitridation (in first stage) at 550 °C (**a**) 2 min, (**b**) 5 min, (**c**) 10 min (Al/NH_4_Cl = 1:1, applied power: 1200 W).

To evaluate the effectiveness of using plasma as a pre-synthesis process, the experiment was conducted with 1200 W of applied power and 550 °C operational temperature for 2 to 10 min with an Al/NH_4_Cl ratio = 1:1. XRD patterns of AlN/Al powder using plasma pre-synthesis nitridation showed that weak AlN crystal peak intensity could be formed by plasma-nitridation for 2 to 10 min (Figure 5). The characteristic peak intensity ratio, AlN (2θ = 33.2)/Al (2θ = 44.8), increased from 0.13 (at 2 min) to 0.16 (at 10 min). The results also indicated that even at a temperature (550 °C) lower than the thermal chemical nitridation temperature (about 900 °C) or melting point of Al metal (660 °C), Al particles still formed an AlN shell on the surface of the Al powders using microwave discharge due to the formation of high energetic N-containing species that can react with Al. Once the AlN thin shell was formed to avoid the aggregation of melting Al, the following high temperature thermal nitridation could be carried out for mass production.

**Figure 6:**
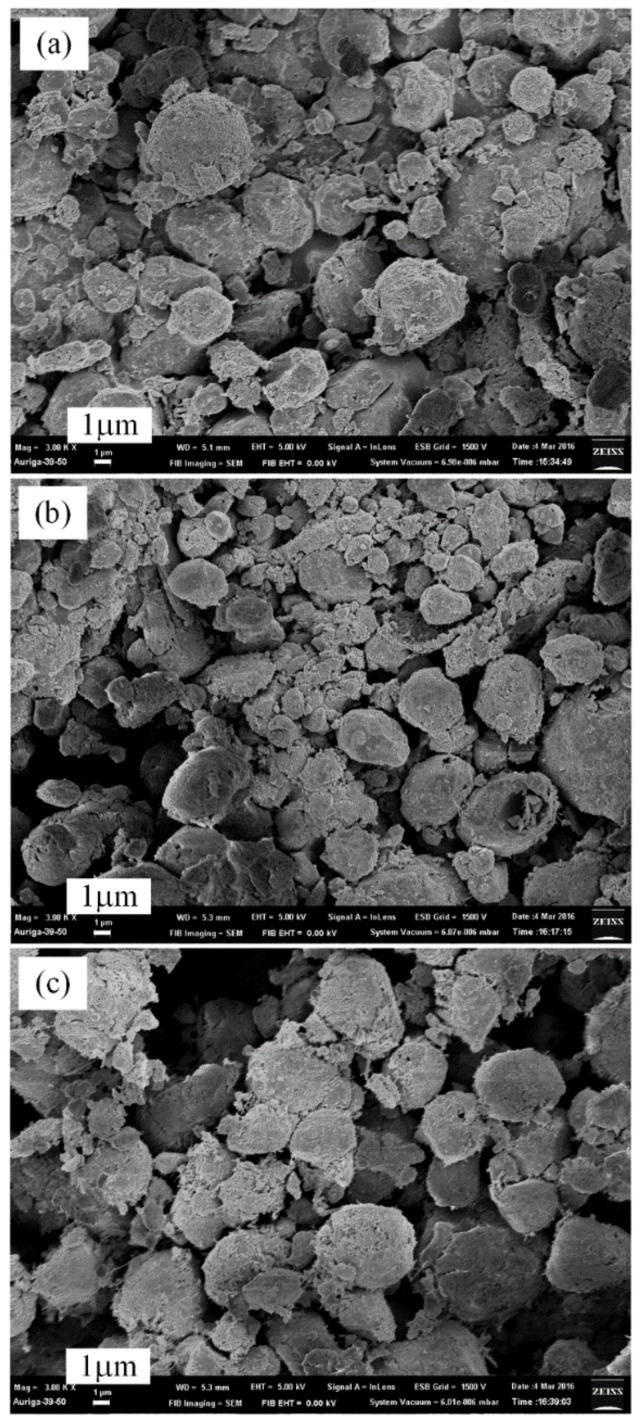
SEM micrographs (3000×) of pre-synthesized AlN/Al powders by plasma nitridation technique at 550 °C for (**a**) 2 min, (**b**) 5 min, and (**c**) 10 min (Al/NH_4_Cl = 1:1, applied power: 1200 W).

The surfaces of AlN/Al powders produced by the plasma pre-synthesis process for 2–10 min were also examined by SEM to determine their particle sizes and morphologies. As shown in the images (Figure 6), the pre-synthesized AlN/Al powders have a similar size, about 2−5 μm, which is similar to that of the original Al powders. However, the surface of the AlN/Al powders was rougher (Figure 6) than those of original Al particles, which resulted in an increase in surface roughness which raises the overall reaction area, reducing the diffusion resistance and decreasing the nitridation time. 

Through the thermal synthesis process, the formation of AlN is an exothermic reaction by which aluminum metal is reacting with nitrogen: 2Al(s/l) + N_2_(g) → 2AlN(s) + heat at a temperature above 900 °C. The free energy was the most important factor for the formation of AlN, not only internally but also on the AlN surface. N_2_ gases need to overcome the diffusion resistance for passing through the AlN shell into the Al core. However, in the discharge environment, highly active and energetic species, such as excited nitrogen molecules or atoms and nitrogen ions, could be identified in an optical emission spectrum [25,26]. These energetic N-containing species can thermodynamically and easily react with Al atoms to form AlN and shorten the reaction time [27] through the following reaction. 

Acitve N-containing species (excited N_2_, N, N_2_^+^, N^+^ ..) + Al (or Al^3+^) → AlN + ... 

Hence, AlN can be produced at a relative low temperature on the surface of Al powders to form the AlN shell. 

### 3.3. Thermal Nitridation in Second Stage (after Plasma Nitridation) for Synthesizing AlN Powders 

**Figure 7:**
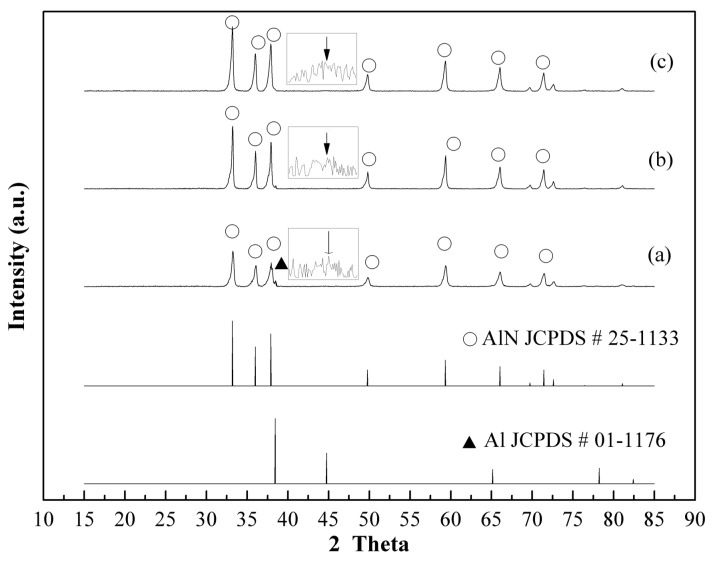
XRD patterns of AlN/Al powders prepared by the plasma pre-synthesis process (1.2 kW, Al/NH_4_Cl = 1/1, and 550 °C for 2 min) followed by thermal nitridation at (**a**) 850 °C, (**b**) 950 °C, (**c**) 1050 °C (heating rate: 15 °C/min, held for 1 h).

When the pre-synthesis of the AlN shell was carried out by a plasma nitridation process for two minutes under the conditions of 1.2 kW, Al/NH_4_Cl = 1:1, and 550 °C, and then followed by the thermal nitridation process with the temperature at 850 °C~1050 °C for 1 h (heating rate: 15 °C/min), the AlN conversion elevated. From the XRD pattern of the synthesized AlN/Al powders, the characteristic peak intensity ratio, AlN (2θ = 33.2°)/Al (2θ = 44.8°), reached 54.8 at 850 °C (Figure 7a), which is higher than the 38.4 by thermal nitridation. Moreover, without Al, the peak can be found with the nitridation temperature being 950 °C or 1050 °C (Figure 7b,c).

According to the evaluation of TGA results for the powders produced by plasma-thermals (550 °C, 2 min) followed by thermal nitridation (950 °C, 1 h) showed that the AlN conversion reached 96%. This is much higher than by only thermal nitridation at 1050 °C and the same nitridation time. As a result, combining a plasma pre-synthesis process and a thermal nitridation stage can provide a high efficiency AlN conversion in comparison to high temperature thermal nitridation, as well as being beneficial to mass production through reductions in nitridation temperature and time. 

**Figure 8:**
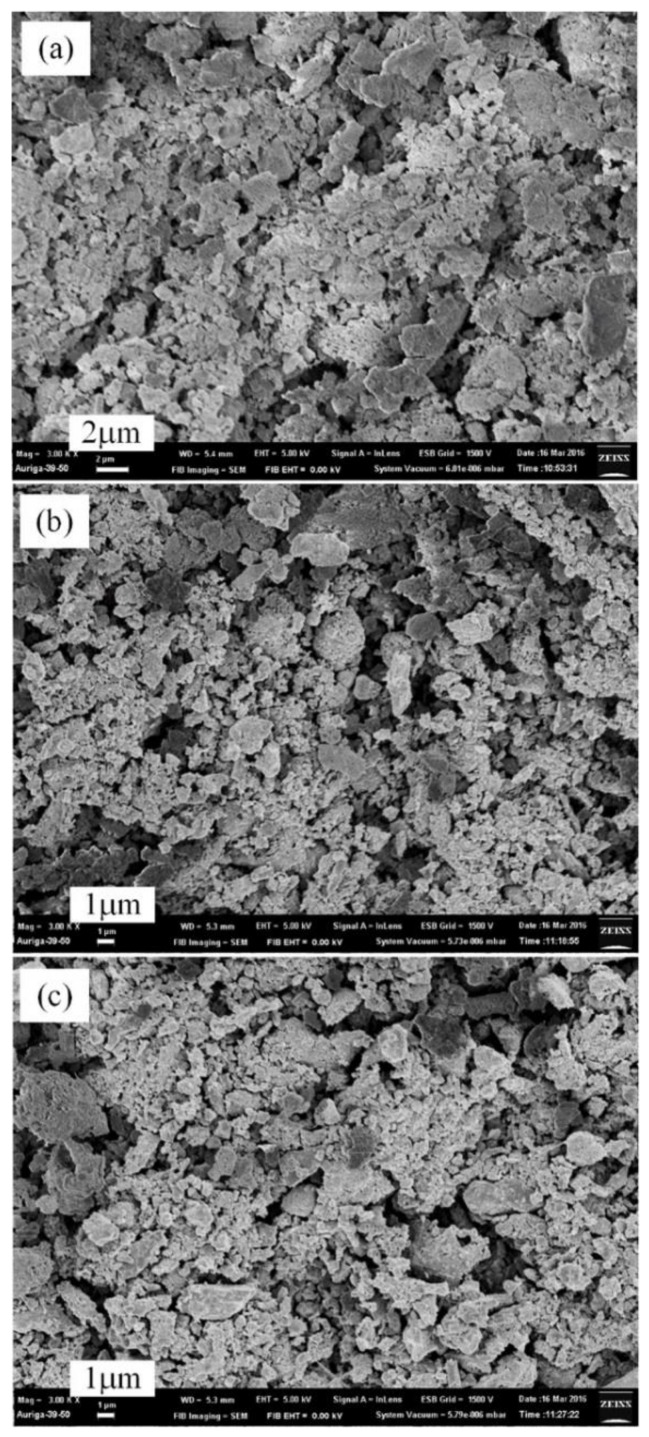
SEM micrographs of powders synthesized by plasma nitridation-thermal nitridation process at (**a**) 850 °C, (**b**) 950 °C, (**c**) 1050 °C in second stage (heating rate: 15 °C/min, held for 1 h).

The SEM images of products synthesized by the two stages of plasma nitridation (at 1.2 kW, Al/NH_4_Cl = 1/1, 550 °C for 2 min) and thermal nitridation (at 850–1050 °C for 1 h) are shown in Figure 8. The diameter of the AlN powders was reduced to about 2–4 μm at 850 °C (Figure 8a). At 950 °C of thermal nitridation temperature, the AlN particle size becomes finer and is less than 2 μm (Figure 8b) with no signs of agglomeration. More fine particles were found when the temperature was raised up to 1050 °C (Figure 8c) with high dispersibility. The results show that initially using a low-temperature plasma as the pre-synthesis process could generate an AlN shell to avoid the flow and aggregation of melting Al metals. Then, the mass production of AlN powders can be carried out rapidly, not only at a high-temperature thermal nitridation process but also without agglomeration being found. 

**Figure 9:**
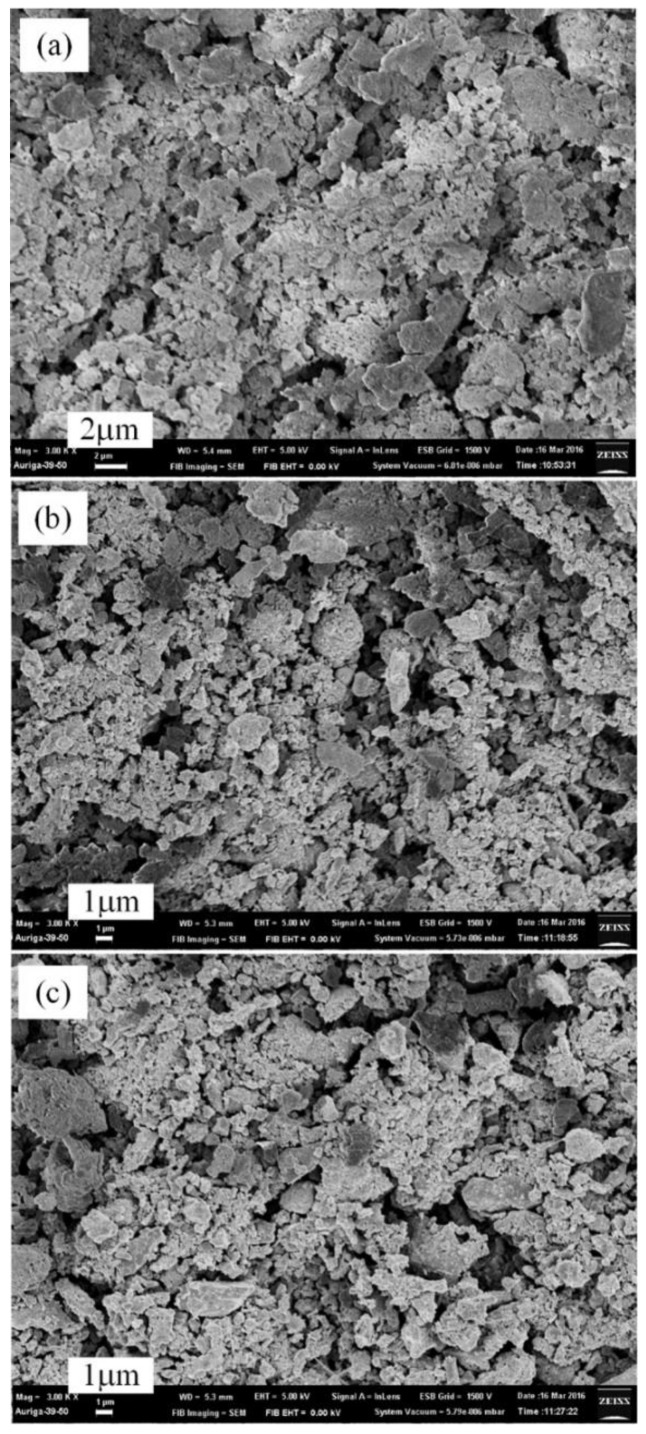
Comparisons of surface characteristics (10,000×) (**a**) original Al powders, (**b**) powders prepared by thermal nitridation at 850 °C and Al/NH_4_Cl = 1/1, (**c**) powders produced by plasma nitridation at 550 °C, 1.2 kW for 2 min and Al/NH_4_Cl = 1:1.

The surface characteristics of Al and synthesized AlN/Al powder were compared. Figure 9 shows the SEM images of the original Al powder (a), AlN/Al powders produced by the thermal nitridation process at 850 °C (Al/NH_4_Cl = 1:1, heating rate: 15 °C/min, held for 0 h) (b), and AlN/Al powers prepared by plasma nitridation at 550 °C (Al/NH_4_Cl = 1:1, 1.2 kW, 2 min) (c). The original Al powders show very dense and smooth surfaces (Figure 9a), while a slightly rough surface with very few pores can be found on the powders produced through the thermal nitridation process (Figure 9b) due to the very low nitridation rate. However, the surface of particles treated by the plasma pre-synthesis technique tended to be rougher and presented more small porosity on the surfaces surrounded by nanowires (Figure 9c). This porosity would allow nitrogen gas diffusion to occur faster, facilitating the nitridation process, and significantly promoting the AlN conversion by keeping the particle size equal or less than that of the original Al particles. In other words, the nitridation efficiency of Al surfaces and mass transfer rate by plasma pre-synthesis technique is much higher than that of the traditional thermal nitridation process. All these characteristics indicate that using plasma techniques as a pre-synthesis process can effectively resolve the agglomeration issue encountered during the traditional thermal nitridation process. 

## 4. Conclusions

In industrial manufacturing, the thermal nitridation technique is commonly used to produce AlN powder from Al powders, despite the need to operate at high-temperatures (>1000 °C) for long reaction times (3–6 h). This is due to the occurrence of aggregation, which has resulted in the expansion of synthesized AlN/Al particle sizes and reduced the AlN yield. Mass production of AlN particles at a low nitridation temperature with short reaction times through the use of plasma sees two major benefits. It not only generates a large amount of active species at 550 °C but also avoids melting Al metal as a pre-synthesis process for 2–10 min to form the AlN shell layer with many pores. Afterwards, utilizing the thermal nitridation process at 850 °C or 950 °C for 1 h can synthesize a high level of purity within the AlN particles by keeping the particle size smaller than that of the original Al particles with great-dispersibility Essentially, the two-stages of plasma nitridation and thermal nitridation have excellent potential as a synthesis process for fine AlN particles and are especially suited for use in for mass production.
